# Mining for the antibody-antigen interacting associations that predict the B cell epitopes

**DOI:** 10.1186/1472-6807-10-S1-S6

**Published:** 2010-05-17

**Authors:** Liang Zhao, Jinyan Li

**Affiliations:** 1 Bioinformatics Research Center, & School of Computer Engineering, Nanyang Technological University, Nanyang Avenue, Singapore 639798; 2 Bioinformatics Research Center, & School of Computer Engineering, Nanyang Technological University, Nanyang Avenue, Singapore 639798

## Abstract

**Background:**

Predicting B-cell epitopes is very important for designing vaccines and drugs to fight against the infectious agents. However, due to the high complexity of this problem, previous prediction methods that focus on linear and conformational epitope prediction are both unsatisfactory. In addition, antigen interacting with antibody is context dependent and the coarse binary classification of antigen residues into epitope and non-epitope without the corresponding antibody may not reveal the biological reality. Therefore, we take a novel way to identify epitopes by using associations between antibodies and antigens.

**Results:**

Given a pair of antibody-antigen sequences, the epitope residues can be identified by two types of associations: paratope-epitope interacting biclique and cooccurrent pattern of interacting residue pairs. As the association itself does not include the neighborhood information on the primary sequence, residues' cooperativity and relative composition are then used to enhance our method. Evaluation carried out on a benchmark data set shows that the proposed method produces very good performance in terms of accuracy. After compared with other two structure-based B-cell epitope prediction methods, results show that the proposed method is competitive to, sometimes even better than, the structure-based methods which have much smaller applicability scope.

**Conclusions:**

The proposed method leads to a new way of identifying B-cell epitopes. Besides, this antibody-specified epitope prediction can provide more precise and helpful information for wet-lab experiments.

## Background

Secreted antibody plays a critical role in humoral immune responses. These antibodies protect the normal cellules or tissues from invaders and infected self cells by neutralizing them through interacting with the pathogenic agents. Subsequently, the neutralized cells are eliminated by scavenger cells, such as macrophage. During this process, antibody interacting with antigen is a fundamental and essential step in immune response. Hence, identifying the set of residues within antigen which are recognized by a specific antibody is pivotal for understanding the mechanism behind antibody-antigen interaction. Consequently, this knowledge in antibody-antigen interaction will shed new light on vaccine design, disease therapy and so on [1].

The small set of residues within antigen sequence that can be recognized by antibody is named as epitope [2]. Epitopes can be categorized into two types: continuous and discontinuous [3]. A continuous/linear epitope is a stretch of consecutive residues in the primary sequence that can bind to a specific antibody, while a discontinuous/conformational epitope is comprised of stretch of residues that are far away from each other in the primary sequence but are brought to spatial proximity as a result of polypeptide folding. Accordingly, a paratope is the part of residues within antibody that interact with the corresponding antigen. Due to the importance of identifying epitopes within antigen, many researchers have devoted themselves to this area.

Intensive efforts have been made to predict epitopes based on physico-chemical properties of antigen interacting with antibody, particularly focus on linear epitope prediction due to its relatively lower complexity. For example, the hydrophilicity scale information of the individual amino acids [4] was adopted by Parker* et al.*, and the flexibility of epitope sequences was used by Karplus* et al. *, to predict linear epitopes [5,6]. The relative accessible surface area of each residue and the three dimensional structure information on antigen sequences were combined together by Kulkarni-Kale* et al.* to predict the conformational epitopes [7]; and the exposure area, amino acids statistical significance and spatial information were utilized by Andersen* et al.* to predict the conformational epitopes as well [8]. Besides, other features, such as polarity [9] and antigenic propensity [10] were also considered to cope with this prediction problem. However, the prediction results are far from satisfied. For example, the performance of the propensity scale based methods are only slightly better than the random projection method [11,12], and it does not improve much even after structural information is added [13].

Several reasons can be used to explain this intractable problem. First of all, epitopes highly depend on specific type of antibody that can recognize them, and most of the antigen surface residues may be antigenic when it is exposed to different circumstances. Therefore, epitope prediction based on binary classification may not reveal the biological reality [14]. Unfortunately, all the aforementioned methods only focused on antigens and overlooked the antibody-antigen relationships. Second, antigen itself is very complicated, and it can range from a few residues to a very large protein. However, epitope residues only take a small portion of the entire antigen residues, thus it is an anomaly detection problem. Third, although the residues that constitute the epitopes are rare, they should cooperate with each other rather than appear independently [15]. However, all the properties that have been used are residue-independent, and only a few methods consider the effect from the neighborhood residues [16].

To overcome these obstacles for a better understanding of antibody-antigen interaction, we propose a novel method to predict epitopes based on associations between antibody-antigen interactions. The intuitive reasons of identifying epitope by associations are: (i) associations not only address the contextual dependence between antibody and antigen, but also reveal the spatial relation within the contact residues; (ii) epitope prediction is very difficult while paratope identification is much easier, therefore linking antibody and antigen together will bridge over the gap; (iii) many research findings have corroborated that paratope-epitope interaction has a tight complementary relationship [17,18], thus it is plausible to link antibody and antigen together. This lock-and-key relationship is utilized in a novel way in this work to capture structural associations between epitopes and paratopes that are then used to predict epitopes in antibody-antigen interacting complexes. Another observation is that paratopes are mainly located in the six complementarity determining regions (CDRs) in an antibody [19], namely L1, L2, L3, H1, H2 and H3. L1, L2 and L3 are from the antibody light chain, while H1, H2 and H3 come from the antibody heavy chain. Therefore, it is relatively very easy to identify paratope residues.

The proposed method is dubbed as** Bepar** which is a short form for** B**-cell** e**pitope** p**rediction through **a**ssociation** r**ules. Our method is trained on antibody-antigen interacting PDB data, and it can be applied to any antibody-antigen sequence pair. The key idea of our method is the detection of association patterns between antibody and antigen residues that can unveil the contextual dependence of the binding site, meanwhile can delineate the residues' spatial relation within the paratope and epitope. As the association idea alone does not involve the neighborhood information in the primary sequence, we integrate the residue's one-side cooperativity to strengthen our method. Furthermore, amino acid's relative composition within the paratopes and epitopes is also calculated to provide a more detailed and precise portrait for epitope prediction as well.

## Methods

### Data preparation

A benchmark data set consists of 82 antibody-antigen complexes that had been constructed by Ponomarenko* et al.*[13] is adopted in this work. The structural complexes of this data set had been manually examined against IEDB [20], and the duplicate complexes had been eliminated as well. In order to improve the accuracy of modeling, the resolution of all complexes have been required to be less than or equal to 3Å. Besides, the protein complexes whose paratope residues mainly situated outside of six CDRs are excluded from the data set. Following these pre-processes, remaining 59 antibody-antigen complexes are used for conducting our experiment. Our method's performance is evaluated based on these 59 complexes by using leave-one-out cross validation.

### Epitopes and paratopes in our training data

Given an antigen-antibody PDB [21] complex, a distance threshold of 4Å is used to determine the epitope residues and paratope residues from the contact residues. This threshold is recommended by [8] as it has been reported that it can capture the epitopes with a high precision. The distance is calculated in Euclidian space between two atoms, except hydrogen, where one atom is from an antigen residue and the other one is from an antibody residue. If the distance is not larger than this threshold, then they will be considered. The involved residue that comes from antigen is named an epitope residue, while the residue comes from antibody is denoted as a paratope residue.

### Amino acid's relative composition and cooperativity calculation

The six CDRs of an antibody can be easily identified by using the Chothia CDR definition [22] which is presented in Table 1, thus paratope residues' relative composition within six CDRs can be calculated by equation (1):

 (1)

where* R_ij_* represents the relative composition of paratope residue* j* in CDR *i*, and* P_ij_* is the statistical composition of residue* j* over the paratope residues in CDR *i*, and* Q_ij_* is the composition of residue* j* against all the residues in CDR* i.*

**Table 1:** Chothia CDR definition

CDR type	CDR range
L1	L24 – L34
L2	L50 – L56
L3	L89 – L97
H1	H26 – H32
H2	H52 – H56
H3	H95 – H102

Similarly, epitope residues' relative composition is computed by equation (2):

 (2)

where* R_j_* represents the relative composition of epitope residue* j*, and* P_j_* is the composition of residue* j* over the whole epitope residues, and* Q_j_* is the composition of residue* j* against all the residues in antigen sequence.

The difference of calculating paratope and epitope residues' relative composition is originated from the fact that paratope residues are mainly located in six CDRs while the arbitrary residues within antigen surface could be antigenic. The definition we adopted to compute residues' relative composition not only considers the contribution of each residue in antibody-antigen interaction by its composition, but also includes the significance of each involved residue through the log odd ratio.

With regard to residues' cooperativity, it is defined as a ratio between an individual residue's composition in paratope/epitope over its native composition within antibody/antigen sequence. Paratope residues' cooperativity is given by equation (3):

 (3)

where* C_i,jk_* represents the cooperativity of paratope residues* jk* within CDR i,* P_i,jk_* is the composition of contiguous residues* jk* over the paratope residues in CDR *i*, and* Q_i,jk_* is the composition of residues* jk* in all the residues in CDR* i.*

In the same way, epitope residues' cooperativity is defined by equation (4):

 (4)

where* C_jk_* represents the cooperativity of epitope residues* jk, P_jk_* is the composition of contiguous residues *jk* over the epitope residues in antigen sequence, and* Q_jk_* is the composition of residues* jk* over all the residues in antigen sequence.

Residues' relative composition is used to identify seed paratope/epitope residues, while residues' cooperativity aims at screening out the neighborhood paratope/epitope residues. This two stages detection can enhance the capability of epitope identification.

### Mining paratope-epitope interacting bicliques and cooccurrent patterns of interacting residue pairs

The associations between an epitope and a paratope is described by paratope-epitope residues interacting biclique and a cooccurrent pattern of paratope-epitope interacting residue pairs.

Interacting biclique is a subgraph* G* = 〈{*V*_1_,*V*_2_}, *E* 〉. Here *V*_1_ is a set of paratope residues,* V*_2_ is a set of epitope residues, and* E* is the set of interactions that , and ∀*v*_1_ ∈ *V*_1_, ∀*v*_2_ ∈ *V*_2_, 〈v_1_, *v*_2_〉 ∈ *E*. Two nodes* v*_1_ and* v*_2_ can form an edge (or say that the residues* v*_1_ and* v*_2_ are interacting) if and only if there exists at least one pair of atoms' distance, except hydrogen, between* v*_1_ and* v*_2_ that is not larger than 4Å.

Interacting bicliques are detected by the following steps: (i) convert an antibody-antigen interacting complex into a bipartite graph, where the vertices are the paratope and epitope residues and the edges are the contact residue pairs; (ii) translate the bipartite graph into a set of transactions with the idea introduced by Li and Liu [23] which builds a connection between bipartite graph and transactions. That is, each bipartite graph is a set of transactions, each transaction ID is an unique epitope residue, and the items within this transaction are the paratope residues that interact with this distinctive epitope residue; (iii) mine all the frequent bicliques from this set of transactions by LCM [24] which is an efficient algorithm to mine frequent item set from a transactional data base and (iv) calculate the statistical frequency of each biclique that appears in different complexes, and filter the frequent bicliques with a 8% occurrence level or 5% occurrence level but with a no less than three times redundancy. The redundancy here means the average count of each individual bicliques that appear in one complex.

Cooccurrent pattern of interacting residue pair is a pattern of two sets of interacting residue pairs, in which if one set occurs in the antibody-antigen interacting complex then the other one will also appear in the same complex with a particular probability (or, confidence level). The mathematical form is given by: . 〈*p* : *e* 〉 is an interacting residue pair with* p* representing a paratope residue and* e* an epitope residue. The left part of the cooccurrent pattern is a set of frequent interacting residue pairs and the right part is a set of cooccurrent interacting residue pairs. Given a set of antibody-antigen interacting PDB complexes, the cooccurrent patterns of interacting residue pairs can be detected through the following three steps. At first, determining all the interacting residue pairs from antibody-antigen complexes by using a distance threshold of 4Å. Second, translating all the interacting residue pairs from each complex into a transaction, thus the number of transactions equals to the number of complexes. The item in each transaction is a unique integer which is mapped by  Where* I_p_* is a paratope residue index and* I_e_* is an epitope residue index. This index is determined by Kyte and Doolittle's hydropathy index order [25]. In the last step, cooccurrent patterns of interacting residue pairs are identified by an association rule mining software implemented by [26]. The significant cooccurrent pattern of interacting residue pairs are remained if they have a ≥ 10% support level and ≥ 80% confidence level.

Interacting bicliques capture the relation between paratope residues and epitope residues which can address the context dependent issue between antibody and antigen, meanwhile cooccurrent patterns of interacting residue pairs will span this relation between interacting residue pairs. This reciprocal consolidation can provide an accurate performance on epitope prediction.

### Epitope prediction by associations

**Figure 1:**
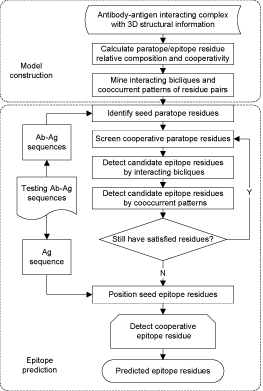
**Flowchart** Flowchart of model construction and epitope prediction

In order to predict epitopes, the prediction model should be constructed on antibody-antigen structural complexes first, and then it is applied to predict epitopes from antibody-antigen sequences without 3D structural information. A flow chart of the processes is shown in Figure 1. The model construction components have been described in the immediate previous two subsections, i.e. calculating paratope/epitope residue's relative composition and cooperativity, and mining interacting bicliques and cooccurrent patterns of interacting residue pairs. From now on, we devote ourselves to the epitope prediction modules. Given an antibody-antigen sequence pair, the antibody heavy chain and light are numbered by modified-Chothia numbering scheme [27] first, then six CDRs are determined by Chothia CDR definition [22], subsequently epitope residues are identified through the steps described as follows:

#### Identifying seed paratope residues

Given the six CDRs of antibody, the seed paratope residues can be identified by using paratope residues' relative composition that generated in the model construction stage. More exactly, each residue within six CDRs is examined one-by-one according to the CDR dependent residue's relative composition threshold *T_R_.* One residue is marked as paratope residue if its relative composition is ≥* T_R_.* Usually, only the top three residues will pass this test.

#### Screening cooperative paratope residues

Based on the seed paratope residues, the cooperative paratope residues can be picked out by using paratope residues' cooperativity. It is achieved by scanning the cooperativity between each seed paratope residue* i* and its neighborhood residues against paratope residues' cooperativity threshold. Once the cooperativity between residue* i* and its neighbor* j* is larger or equals to the preset threshold then residue* j* is assigned as paratope residue. Paratope/epitope residues usually cooperate with each other, therefore the search space of neighborhood residues are restricted in [*i* – 2,* i* + 2] for a given seed residue *i*. Both the left neighbors and the right neighbors should be within the same CDR as *i* 's. Empirically, the top ten per cent of cooperative residues are considered as paratope residues.

#### Detecting candidate epitope residues by interacting bicliques

A subset of paratope residues can be identified through the first two steps. In this step a partial candidate epitope residues can be specified by using interacting biclique which is served as a bridge to link paratope and epitope residues together. Exactly, all the paratope residues from each interacting biclique are checked against the pre-identified paratope residues, and one interacting biclique is believed to appear in this complex if all the paratope residues have been found in the pre-identified set of paratope residues, subsequently the epitope residues within this interacting biclique are considered as candidate epitope residues.

#### Detecting candidate epitope residues by cooccurrent patterns of interacting residue pairs

Part of candidate epitope residues can be identified by paratope-epitope interacting bicliques, however interacting biclique can only reveal the local relation between paratope and epitope residues. Hence cooccurrent pattern of interacting residue pairs is used to span the correlation between interacting residue pairs.

For each cooccurrent pattern, the left part of the pattern (or the frequent interacting residue pairs) is checked against the already identified paratope-epitope interacting residue pairs. If all the interacting residue pairs from the frequent part of the cooccurrent pattern have been picked out already, then the right part of this pattern is considered as implied interacting residue pairs in the same complex. The residues from this implied interacting residue pairs are added to paratope and candidate epitope respectively to broaden the search space.

The immediate above three Steps will repeat until satisfied paratope and epitope residues cannot be found anymore. Following the above steps, the candidate epitope residues can be confirmed and their positions are localized by the following two steps.

#### Positioning seed epitope residues

A candidate epitope residue is confirmed as an epitope residue if its relative composition meets the preset threshold. This process is conducted along the whole antigen sequence to localize the seed epitope residues. Empirically, the top four epitope residues are selected in terms of epitope residues relative composition.

#### Detecting cooperative epitope residues

Based on the seed of epitope residues, the cooperative epitope residues can be determined by using epitope residues' cooperativity. One residue is assigned as a cooperative epitope residue if the cooperativity between this residue and the seed epitope residue is larger or equals to the predefined cooperativity threshold. In this work, only the residues' cooperativity within the top ten per cent is considered. This process will be terminated when no satisfied neighborhood epitope residues can be identified again. Through the above six steps, we can identify epitope residues with a high accuracy. We note that seed epitope residue identification takes the candidate epitope residues into consideration, while cooperative epitope residues detection overlooks this constraint. There are two reasons to explain this strategy: first, associations (paratope-epitope interacting biclique and cooccurrent pattern of interacting residue pairs) can only capture the significant paratope and epitope residues instead of the complete paratope and epitope residues; and second, the looseness constraint of cooperativity applied on seed epitope residues can generalize the prediction. These two aspects guarantee the prediction model with a good performance.

## Results and discussion

### Residues relative composition and cooperativity in epitope and paratope

**Figure 2:**
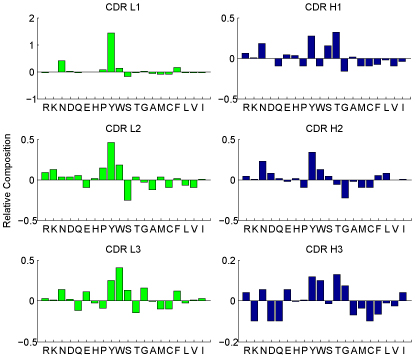
**Paratope relative composition** Paratope residues' relative composition in six CDRs

**Figure 3:**
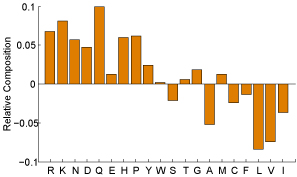
**Epitope relative composition** Epitope residues' relative composition

Paratope and epitope residues' relative composition are shown in Figure 2 and Figure 3 respectively. It is clear that each residue makes remarkably dissimilar contribution in antibody-antigen binding. On the other hand, the same residue has diverse preferences in the six CDRs. For example, paratope residues Y, W, N and R make a great contribution in antigen binding, however this observation does not hold for the epitope residues' relative composition, especially for residues Y and W. Besides, paratope residue T is over expressed in CDR H1 and H3 while residue S is up regulated in CDR H1 and L3. For epitope residues, the values shown in Figure 3 precisely illustrate that epitope residues prefer hydrophilic residues to hydrophobic residues. The observations derived from the profile of epitope residues relative composition shown in Figure 3 just partially agree with the findings reported in [28]. That is, epitope residues are enriched with charged and polar residues and significantly depleted in hydrophobic residues. The difference is narrowed down to residues Y and W. Rubinstein* et al.*[28] argued that epitopes were significantly over expressed by residues Y and W, but this observation is not so significant in our result. The reason should be that residues Y and W are indeed enriched in epitopes but they also have relatively very high composition in antigen sequences. Nevertheless, our observations are supported by the findings on antibody-antigen interaction explored by Jackson [29] and also applauded by the observations reported in [30]. These observations corroborate our idea of treating the six CDRs separately.

**Figure 4:**
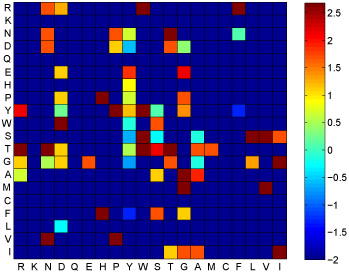
**H3 cooperativity** Paratope residues' cooperativity in CDR H3. Value is post-modified by logarithm and –∞ is replaced by -2

**Figure 5:**
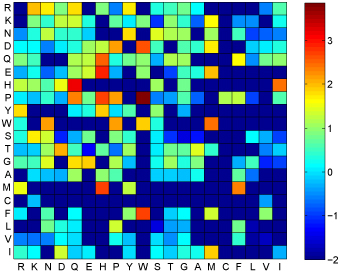
**Ag cooperativity** Epitope residues' cooperativity. Value is post-modified by logarithm and –∞ is replaced by -2.

With regard to residues' cooperativity, paratope residues' cooperativity in CDR H3 which makes the most contribution in antigen binding [31,32] is shown in Figure 4 and epitope residues' cooperativity is shown in Figure 5. Arguments also have been made somewhere that epitope residues tend to act cooperatively [33]. According to the residues' cooperativity shown in these two figures, we can find that paratope residues usually cooperate with Y, W, S, T and G while epitope residues prefer pairs of hydrophilic residues. Interestingly, hydrophobic residues are scarce in paratope, but once they appear in paratope then they tend to cooperate with the particular residues.

The values shown in Figure 4 and Figure 5 are calculated in terms of 1-free connectivity, i.e. at most one non-paratope/epitope residue is allowed to insert between two paratope/epitope residues. Similarly, 0-free connectivity means non-paratope/epitope residue insertion within two paratope/epitope residues is unacceptable. Although epitopes are categorized into linear and conformational epitope, most part of the conformational epitope is constituted by some consecutive residues [3]. Therefore it is reasonable to search the neighborhood residues one more position beyond its immediate neighbors.

**Figure 6:**
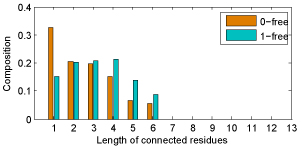
**Paratope connectivity** Paratope residues' connectivity with respect to 0-free and 1-free

**Figure 7:**
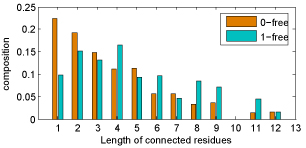
**Epitope connectivity** Epitope residues' connectivity with respect to 0-free and 1-free

Paratope residues connectivity is shown in Figure 6, and epitope residues connectivity is shown in Figure 7. From the results shown in these two figures we can see that if 1-free connectivity is used then the composition of separated paratope/epitope residue deceases significantly. More exactly, the isolated paratope residue composition drops from 32.7% to 15.2%, and the separated epitope residue composition also decreases from 22.4% to 9.7%.

### Interacting biclique and cooccurrent pattern of interacting residue pairs in antibody-antigen complex

The interacting biclique captures the close relationship between one set of paratope residues and the other set of epitope residues, thus it can address the context dependent issue within antibody and antigen interaction. The top ten frequent interacting bicliques are listed in Table 2. It is not out of expectation that one-versus-one bicliques are ranked as the most frequent ones. Although this seems trivial, it could offer a great help especially when not enough paratope residues can be identified in the early stages. The results reveal that the paratope residues are enriched with residue Y and epitope residues are rich of residues R and K. These observations are also in accordance with the findings given by residues' relative composition. The cooccurrent patterns of interacting residue pairs with support level (or frequency) ≥ 10% and confidence level (or probability) ≥ 80% are remained in this work to span the correlation between paratope and epitope. These thresholds are chosen empirically by both considering the model's reliability and compatibility. The cooccurrent patterns with 100% confidence level are presented in Table 3. In comparison with the involved residues shown in Table 2, conclusion can be drawn that cooccurrent pattern includes much broader residues which indeed can broaden the paratope and epitope search space. Interacting biclique and cooccurrent pattern can benefit reciprocally. In the one hand, interacting biclique is a little stringent but provides relatively more precise interacting residue pairs for identifying cooccurrent residue pairs; in the other hand, cooccurrent pattern of interacting residue pairs is lesser strict and can broaden the search space of paratope and epitope residues which in turn will benefit interacting biclique specification.

**Table 2:** Top ten frequent association (bi-cliques) from antibody-antigen interacting complexes

No.	Ab. ^1^	Ag.^2^	Frequency	Redundancy
1	D	K	22.0% (13/59)	1.92 (25/13)
2	Y	E	18.6% (11/59)	2.45 (27/11)
3	Y	N	16.9% (10/59)	1.80 (18/10)
4	S	E	16.9% (10/59)	1.70 (17/10)
5	Y	K	15.3% ( 9/59)	1.78 (16/9)
6	N	R	15.3% ( 9/59)	1.44 (13/9)
7	Y	R	15.3% ( 9/59)	3.56 (32/9)
8	D,Y	K	15.3% ( 9/59)	1.78 (16/9)
9	S,Y	Q	13.6% ( 8/59)	1.75 (14/8)
10	G	R	13.6% ( 8/59)	4.38 (35/8)

^1^Antibody residue(s).

^2^Antigen residue(s).

**Table 3:** Co-occurrent epitope-paratope interacting residue pairs with support level larger than 10%

No.	FIRP.^1^	CIRP.^2^	Confidence
1	〈Y:K〉, 〈S:Q〉	〈Y:Q〉	100%
2	〈Y:G〉, 〈W:K〉	〈Y:K〉	100%
3	〈Y:N〉, 〈W:K〉	〈Y:K〉	100%
4	〈N:N〉, 〈W:K〉	〈Y:K〉	100%
5	〈S:Q〉, 〈Y:F〉	〈Y:Q〉	100%
6	〈Y:K〉, 〈D:N〉	〈Y:N〉	100%
7	〈Y:G〉, 〈Y:Y〉	〈Y:K〉	100%
8	〈W:K〉, 〈Y:Y〉	〈Y:K〉	100%
9	〈Y:G〉, 〈T:K〉	〈Y:K〉	100%
10	〈T:N〉, 〈D:S〉	〈D:N〉	100%
11	〈D:N〉, 〈D:S〉	〈T:N〉	100%
12	〈G:Q〉	〈Y:Q〉	100%

^1^ Frequent interaction residue pairs

^2^ Cooccurrent interaction residue pairs

### Prediction performance by Bepar and its evaluation

The performance of Bepar is quantified by means of sensitivity (Sens.), specificity (Spec.), accuracy (Accu.) and area under the carve (AUC). Their definitions are given by the following formulae:

where TP is the number of correctly identified epitope residues, TN is the number of correctly detected non-epitope residues, FP is the number of incorrectly predicted epitope residues and FN is the number of incorrectly speculated non-epitope residues. In these quantifiers, AUC is especially recommended by [14]. Therefore, we also adopt this evaluation matrix to make comparison between our model and other structure-based B cell epitope prediction tools.

In order to avoid the over-fitting problem caused by the self examination (which can overestimate the method's performance), the leave-one-out cross validation is used to evaluate our model. That is, if there are N samples then the evaluation will run N times. In each round, one sample is left out to do and only do prediction and the remaining samples are used to train the prediction model. With regard to our model, each time there are 58 antibody-antigen PDB complexes are used to train the model and 1 antibody-antigen complex without its structural information is considered to test this model. To qualify the prediction capability of our method, we compare the performance of our model with two structure-based B-cell epitope prediction tools CEP [7] and DiscoTope [8]. CEP takes antigen structures as input and predicts epitopes by using residues accessibility and spatial distance cut-off. Similarly, DiscoTope predicts epitopes from antigen structures based on amino acid statistics, spatial information and surface accessibility. The performances on CEP and DiscoTope are obtained from the results conducted by Ponomarenko* et al.*[13]. For convenience, the results generated by CEP are chosen from the average value and the results of DiscoTope are selected from the values with a cut-off threshold of -7.7. Some epitopes cannot be identified by these three methods, therefore the common data with 32 samples are selected to evaluate these three methods. The detailed performances of these three methods are shown in Table 4. Results reveal that Bepar shows competitive performance on epitope prediction even without antigen 3D structure information.

**Table 4:** Prediction results generated by sequence-based model and two structure-based models

				Bepar			CEP			DiscoTope		
						
PDB ID	H^1^	L^2^	Ag^3^	sens.	spec.	AUC	sens.	spec.	AUC	sens.	spec.	AUC
1A14	H	L	N	0.11	0.75	0.43	0.00	0.94	0.47	0.76	0.86	0.81
1AR1	C	D	B	0.27	0.89	0.58	0.13	0.85	0.49	0.00	0.89	0.45
1EO8	H	L	A	0.33	0.61	0.47	0.18	0.89	0.54	0.17	0.78	0.48
1EZV	X	Y	E	0.53	0.74	0.64	0.31	0.63	0.47	1.00	0.76	0.88
1FNS	H	L	A	0.58	0.83	0.71	0.00	0.87	0.44	0.67	0.9	0.79
1FSK	C	B	A	0.59	0.71	0.65	0.12	0.88	0.50	0.76	0.67	0.72
1G9M	H	L	G	0.33	0.75	0.54	0.18	0.88	0.53	0.08	0.79	0.44
1H0D	B	A	C	0.44	0.70	0.57	0.44	0.65	0.55	0.35	0.63	0.49
1IQD	B	A	C	0.31	0.71	0.51	0.07	0.84	0.46	0.56	0.85	0.71
1JPS	H	L	T	0.53	0.74	0.64	0.25	0.83	0.54	0.33	0.85	0.59
1JRH*	H	L	I	0.47	0.71	0.59	0.73	0.32	0.53	0.60	0.73	0.67
1LK3	H	L	A	0.39	0.71	0.55	0.17	0.87	0.52	0.61	0.84	0.73
1MHP^*^	X	Y	B	0.37	0.73	0.55	0.11	0.92	0.52	0.53	0.84	0.69
1NFD	H	G	D	0.33	0.79	0.56	0.25	0.85	0.55	0.77	0.77	0.77
1NL0^*^	H	L	G	0.80	0.39	0.60	0.71	0.84	0.78	0.57	0.82	0.70
1NSN^*^	H	L	S	0.24	0.77	0.51	0.06	0.77	0.42	0.39	0.68	0.54
1OAZ	H	L	A	0.53	0.82	0.67	0.59	0.69	0.64	0.29	0.81	0.55
1ORS	B	A	C	0.50	0.88	0.69	0.78	0.63	0.66	0.00	0.84	0.42
1OSP	H	L	O	0.30	0.64	0.47	0.17	0.82	0.50	0.53	0.80	0.67
1PKQ^*^	B	A	E	0.59	0.64	0.62	0.44	0.68	0.56	0.47	0.79	0.63
1R3J	B	A	C	0.23	0.84	0.54	0.42	0.62	0.52	0.08	0.91	0.50
1RJL^*^	B	A	C	0.42	0.54	0.48	0.58	0.48	0.53	0.54	0.71	0.63
1SY6*	H	L	A	0.82	0.72	0.77	0.30	0.86	0.58	0.91	0.68	0.80
1TQB	B	C	A	0.12	0.66	0.39	0.11	0.71	0.41	0.78	0.36	0.57
1TZI^*^	B	A	V	0.50	0.77	0.64	1.00	0.21	0.61	0.50	0.58	0.54
1V7M*	H	L	V	0.69	0.76	0.73	0.31	0.80	0.56	0.06	0.95	0.51
1WEJ^*^	H	L	F	0.27	0.66	0.47	0.10	0.69	0.40	0.45	0.45	0.45
1YJD^*^	H	L	C	0.43	0.88	0.66	0.36	0.68	0.52	0.21	0.87	0.54
1ZTX^*^	H	L	E	0.25	0.72	0.49	0.75	0.34	0.55	0.19	0.88	0.54
2ADF	H	L	A	0.13	0.62	0.38	0.32	0.88	0.60	0.15	0.97	0.56
2AEP*	H	L	A	0.79	0.71	0.75	0.10	0.93	0.52	0.14	0.85	0.50
2JEL*	H	L	P	0.27	0.74	0.51	0.43	0.45	0.44	0.07	0.94	0.51

^1^ Antibody heavy chain.

^2^	Antibody light chain.

^3^	Antigen chain.

* Fourteen non-overlapping samples.

**Figure 8:**
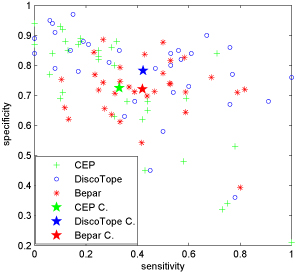
**Performance comparison** Result comparison over the whole common 32 samples that generated by Bepar, CEP and DiscoTope. C. represents the statistic averaged center

Figure 8 visualizes the performances given by Bepar, CEP and DiscotTope in terms of sensitivity and specificity. The averaged over all performance clearly manifest that Bepar makes a great improvement on sensitivity when compared with CEP on the same specificity level, but it presents a lower specificity when compared with DiscoTope with respect to the same level of sensitivity. After investigating the detailed data set, we found that 18 out of 32 samples in the data set applied on DiscoTope are both in the training data and testing data. To compensate this unfairness, the overall statistic averaged results of the evaluation metrics are calculated on the whole data set, the overlapping data set applied on DiscoTope and also the non-overlapping data set respectively. The overlapping data set means the samples both appear in training data and testing data applied on DiscoTope, while the non-overlapping data set represents the samples only appear in testing data. The detailed results are shown in Table 5. It can be seen that Bepar outperforms CEP in every cases according to the AUC values, and it also shows a very competitive performance to DiscoTope when the non-overlapping data set is applied. Hence, we can draw a conclusion that Bepar is a better or at least a competitive candidate B-cell epitope prediction approach even 3D structure is unavailable in the prediction stage.

**Table 5:** Statistic averaged sensitivity and specificity of CEP, DiscoTope and Bepar as well as their standard deviation

Data	Method	Sens.	Spec.	AUC
I ^1^	CEP DiscoTope Bepar	0.33 (± 0.25) **0.42** (± 0.28) 0.42 (± 0.19)	0.73 (± 0.19) **0.78** (± 0.13) 0.72 (± 0.10)	0.53 (± 0.08) **0.60** (± 0.12) 0.57 (± 0.10)

II^2^	CEP DiscoTope Bepar	0.25 (± 0.20) **0.44** (± 0.31) 0.36 (± 0.15)	0.79 (± 0.12) **0.79** (± 0.13) 0.74 (± 0.08)	0.52 (± 0.06) **0.62** (± 0.14) 0.55 (± 0.10)

III^3^	CEP DiscoTope Bepar	0.43 (± 0.28) 0.41 (± 0.23) **0.49** (± 0.21)	0.64 (± 0.23) **0.77** (± 0.10) 0.69 (± 0.10)	0.53 (± 0.09) 0.586(± 0.09) **0.593(±** 0.10)

^1^Common thirty two samples.

^2^ Eighteen overlapping samples which appear both in training data and testing data.

^3^ Fourteen non-overlapping samples which only appear in testing data.

The proposed method Bepar is very novel and promising, but there are still much space for improvement. First, finding the optimal parameters are time-consuming even though empirical parameters can provide a satisfactory result. Second,it is a simple approach to identifying epitopes by their relative compositions and cooperativity based on the candidate epitope residues. Therefore, a sophisticated post-stage prediction method would provide a much better performance.

## Conclusions

B-Cell epitope prediction has attracted increasing attention in the field of immunoinformatics [7,8,33-35]. However, due to its high complexity and scarce structural data sets, such prediction task is full of challenges [11-13]. In this work, we proposed an innovative and efficient method to tackle this problem based on the structural associations between paratopes and epitopes. In comparison to previous structure-based B-cell epitope prediction methods [7,8], Bepar outperforms CEP on every cases in the common data set, and it is also very competitive to DiscoTope when the non overlapping data set is considered. In addition, unlike these two methods, Bepar needs only a relatively small data set with 3D structural information to train the model and can apply to paired sequence data from antibody-antigen complexes.

## Competing interests

The authors declare that they have no competing interests.

## Authors contributions

LZ conceived of the study and drafted the manuscript, JL supervised in the design of the study and helped to finalize the manuscript. All authors read and approved the final manuscript.
